# Risk factors for medication errors in the electronic and manual prescription**^1^**

**DOI:** 10.1590/1518-8345.0642.2742

**Published:** 2016-08-08

**Authors:** Cris Renata Grou Volpe, Eveline Maria Magalhães de Melo, Lucas Barbosa de Aguiar, Diana Lúcia Moura Pinho, Marina Morato Stival

**Affiliations:** 2 PhD, Adjunct Professor, Faculdade de Ceilândia, Universidade de Brasília, Brasília, DF, Brazil.; 3 Undergraduate student in Nursing, Faculdade de Ceilândia, Universidade de Brasília, Brasília, DF, Brasil. Scholarship holder of the Universidade de Brasília, Brasília, DF, Brasil.; 4 PhD, Adjunct Professor, Faculdade de Ceilândia, Universidade de Brasília, Brasília, DF, Brazil.

**Keywords:** Medication Errors, Medication Systems, Electronic Prescribing, Patient Safety

## Abstract

**Objective:**

to compare electronic and manual prescriptions of a public hospital of Brasilia, identifying risk factors for the occurrence of medication errors.

**Method:**

descriptive-exploratory, comparative and retrospective study. Data collection occurred from July 2012 to January 2013, using an instrument for the review of the information contained in medical records related to the medication process. A total of 190 manual and 199 electronic records composed the sample, with 2027 prescriptions each.

**Results:**

compared to the manual prescription, a significant reduction was observed in the risk factors after implantation of the electronic prescription, in items such as "lack of the form of dilution" (71.1% to 22.3%) and "prescription with brand name" (99.5% to 31.5%). Conversely, the risk factors "no check" and "lack of CRM of the prescriber" increased. The lack of the allergy registration and the occurrences related to medication were the same for both groups.

**Conclusion:**

generally, the use of the electronic prescription system was associated with a significant reduction in risk factors for medication errors, concerning the following aspects: illegibility, prescription with brand name and presence of essential items that provide a safe and effective prescription.

## Introduction

The identification of risk factors for errors related to medication administration has proved important to ensure greater safety for patients and health professionals. They can be identified, for example, by analyzing medical prescriptions, which allows preventive actions to reduce the occurrence of adverse events. 

In the literature it can be verified that the electronic prescription (EP) system for medications has enabled higher quality care to hospitalized patients and others involved, demonstrating that the choice of this model can help to reduce errors related to medications by up to 50%^1^ ^-^ ^2^. Other studies also refer to improvements in antibiotic prescriptions and reduced time and cost of hospitalization^3^ ^-^ ^4^. However, there are publications that indicate increased mortality after its implementation^5^ ^-^ ^7^, medical team resistance in the use of electronic prescription due to time constraints, impairments in the interaction between patients and nurses, and lack of integration with the flow work^8^.

These data are worrying, because events of this nature are frequent and constitute a concern for healthcare professionals, patients, and government agencies. Accordingly, the Ministry of Health and the National Health Surveillance Agency (Anvisa) launched, in April 2013, the National Patient Safety Program, with the focus on preventing and reducing the incidence of situations that result in harm to patients.

Internationally, the issue has also been the subject of various investigations. Studies have shown that, after the implementation of the electronic prescription system, there has been a reduction in the frequency of medication errors^1^ ^-^ ^2^ ^,^ ^7^ ^,^ ^9^. In Brazil, however, this strategy has still been little investigated. Among the few studies, one investigated the presence of prescriptions smudged after printing (18%), suspended medications (17%) and lack of information about presentation, time (9%) and route of administration (82%)^10^.

International studies that comparatively investigated manual and electronic prescriptions^11^ ^-^ ^13^, showed a reduction in prescribing error rates and improved outcomes for patients following the implementation of an electronic system. Given the above, knowing the prescription system and its functionality is essential for a safety proposal for both patients and health professionals. 

With regard to the administration of medication, to talk about safety necessarily refers to reducing the risk of errors that generally occur in the prescription, dispensing and administration stages. Studies show that: 72% of them started with the prescription and 15% during the administration^14^ ^).^ In a study conducted recently in Brasilia, Brazil, with a total of 484 doses observed, errors occurred during the administration of the drug in 69.5% of them: 69.6% during the preparation phase, 48.6% were time errors, 1.7% dosage errors and 9.5% derived from omission^15^.

Despite some advantages, one of the difficulties in the adoption of EP may be the high cost of the system, as well as a high risk that its implementation is ineffective and may produce unintended consequences and harm^4^ ^,^ ^16^. In Brazil, various institutions have already adopted this system, however, it is still necessary to know how its implementation is going and how professionals interact with it. It is also fundamental to investigate whether it is, in fact, providing greater safety and quality for the care. 

Considering the potential benefits of the electronic prescription and how the computerized system contributes to reducing medication errors, reducing costs and assuring the quality of care provided, it was proposed to investigate these aspects in a hospital of the city of Brasilia.

Accordingly, this study aimed to compare electronic (EP) and manual prescriptions (MP) of a public hospital of Brasilia, identifying risk factors for the occurrence of medication errors.

## Method

This was an exploratory, descriptive and comparative study, performed in the internal medicine department of a public hospital of Brasilia, the Federal District, Central Region of Brazil. The clinic has 31 beds reserved for patients with chronic and degenerative diseases with long periods of hospitalization, which, because of the treatment, make use of various medications.

It should be noted that in this unit the electronic prescription system was implemented in August 2011. The electronic patient record (EPR) presents the following information: prescription of the medication, request for and results of examinations, notes of nursing professionals, medical evolution and requests for materials and medications. It also contains support for the clinical decision, making the prescription safer and more efficient, namely: allergy alerts, duplicate checking and dose and body mass index (BMI) calculation.

The medical records of patients, aged over 18 years, hospitalized in the internal medicine department, from July 2010 to September 2012, were included. The records not drawn were excluded. The constitution of EP and MP samples was defined by convenience through simple drawing, in order to ensure the inclusion of records that represent admissions for every month of the data collection period. Per month, on average 15 to 17 patients were drawn and included in the analysis list, with the others being excluded. In the event that any of the randomly selected patients were not found, the records of another, in the same month, was drawn.

The MP sample was calculated based on admissions/year in the internal medicine department, this being 456, with an average of 38 per month. Thus, for the calculation of the sample, N= 456, corresponding to the total admissions for the period July 2010 to July 2011, 213 medical records were included to be reviewed, considering a 0.05 confidence level and sampling error of 4%. 

Due to the difficulty of access to the manual patient records after implementation of the electronic system, a sample of 190 patient record manuscripts was analyzed, totaling 2, 027 manual prescriptions (MP), which constituted the MP group. For this collection, the researchers drew up a list of the patient records to be analyzed, which were separated by the medical file sector team and made available to the researchers.

With regard to the EP, total admissions in the internal medicine department from September 2011 to September 2012 were 415. The same sample calculation method was used, giving the number of 200 patient records to be reviewed. As one of the patients had two registration numbers in the system, in the end 199 electronic medical records were analyzed, with a total of 2,027 electronic prescriptions. 

At this time, the patient records were reviewed directly on the computer, after digitizing the patient record drawn and generating the EPR as a PDF.

Data collection was supported by two trained nurses, with retrospective analysis carried out of the information contained in the 4054 prescriptions selected.

The data collected were recorded in a specific instrument, called the Instrument Used in the Review of Patient Records Related to the Medication Process, previously tested and adapted from earlier studies^17^. 

The dependent variables analyzed at this stage were: manual prescriptions (handwritten); electronic prescriptions, performed on the computer and typed into the electronic prescription system. In order to achieve the purpose of assessing the presence or absence of information related to medications in the medical developments and outcomes and nursing notes, these were also analyzed in the medical records included in the sample.

The independent variables were diverse, namely: illegible handwriting, when it was impossible to understand what was written or when at least one item was indecipherable; and legible handwriting, when it was possible to read without difficulty, problems, or spending time trying to understand what was written, which included all the words, numbers, symbols and abbreviations. The absence of the following information was also noted: bed number, registration number, name and CRM of the prescriber, date, registration of allergies, date and time updated, and the lack of data on the presentation of the drug, the route of administration, the form of dilution, the frequency of administration and prescribed medication with brand name. Inappropriate acronyms and abbreviations were considered inadequate, especially abbreviations of drug names, such as HCTZ (hydrochlorothiazide). Further independent variables were: erasures (scratches, smudges, deletion of letters or words of the prescription), scraped or scratched, and changes/suspensions: presence of changes in the prescription throughout the day, change or suspension of the medication or of the care: incomplete time designations, with errors/erasures were also analyzed: lack of information on the medication administration schedule or erasures of the appointed time: medications without checking: lack of registration of the medication administration, the word OK or the symbol "P" corresponding to the administration at the time that the drug was prescribed: lack of justification for not checking: absence of record of justification of the type "does not have in the pharmacy", "missing", "verbally suspended by the physician", in the prescription itself or the outcomes/nursing notes: presence or absence of observations and suspension of medications in the medical or nursing outcomes: and lack of informative record. Finally, the presence or absence of registration of the administration of SOS medications was analyzed in the nursing outcome: SOS medication "if necessary" and "if the need arises". 

The Statistical Package for the Social Sciences (SPSS (r) v. 18. 0) was used to organize and process the data. The categorical variables are reported as absolute and relative frequencies and the numerical variables as mean and standard deviation (minimum and maximum).

All variables were analyzed using univariate and multivariate analyses, considering a significance level of 5%. The odds ratio (OR) was calculated, with confidence intervals (CI) of 95% and, for associations, the chi-square and Mann-Whitney tests were used.

The development of the study complied with the national and international standards of ethics in research involving human subjects, with authorization, under number 017/2012, from the Research Ethics Committee of the Federal District Health Department.

## Results

Table 1 presents the distribution of the manual (MP) and electronic prescriptions (EP).

**Table 1:** Distribution of medical prescriptions according to handwriting, lack of essential items of the prescription, erasures, scheduling and medications without checking. Brasilia, DF, Brazil, 2013

Medical prescription information		Manual n^*^ (%)	Electronic ^*^n (%)	P value^†^	OR^‡^ gross (CI^§^ 95%)
Handwriting					
	Legible	1408(69.5)	2027(100.0)		
	Illegible	88(4.3)	-		
	Partly||	531(26.2)	-		
Lack					
	Bed number	1746(86.1)	1931(95.3)	<0.000	1.57(1.46-1.68)
	Registration number	512(25.3)	204 (10.8)	<0.000	3.02 (2.53-3.60)
	Name and CRM^¶^ of prescriber	24(1.7)	1411(98.3)	<0.000	45.72(30.73-68.03)
	Date	42 (2.1)	4(0.2)	<0.000	10.70 (3.83-29.89)
	Allergies registration	2009(99.1)	1919 (94.7)	0.042	1.13 (1.01-1.27)
	Date and time updated	102 (41.6)	330 (16.3)	<0.000	4.74 (4.10-5.49)
	Justification	1635(80.7)	1865 (92.0)	<0.000	1.51 (1.42-1.61)
Presence					
	Erasures	458 (22.6)	-	-	-
	Changes or suspensions	685 (33.8)	1230 (11.3)	<0.000	1.75(1.65-1.85)
	Incomplete schedule	204 (10.1)	64 (3.2)	<0.000	3.43(2.57-4.57)
	Schedule with error or erasures	293(14.5)	55(2.7)	<0.000	6.05(4.51-8.13)
	Medications without checking**	631(31.1)	1605(79.2)	<0.000	8.41(7.29-9.70)

* n=2027.

† p value.

‡ OR Odds Ratio*.*

§ Confidence Interval

║ Partly. Prescriptions in which it was impossible to completely read all the medications and care prescribed.

¶ Regional Council of Medicine;

** record of performing the procedure in the medical prescription.

Starting from the premise that illegibility increases the risk of medication errors, it was observed that the EP eliminated the occurrence of illegible prescriptions and erasures, which constitutes a major benefit of the system.

 Risk factors related to the lack of date and time updated, registration number and occurrence of allergies were also reduced with the implementation of the electronic system. It was observed that the absence of such information in the MP is significantly greater than in the EP. 

The factor name and CRM of the prescriber was absent in 98.3% of the EP. Importantly, in the case of electronic prescriptions, a password is required to access the system that enables the prescriber to issue prescriptions and make changes; however, the registration data are not recorded in the electronic medical record, which explains the error of absence of prescriber identification information (CRM) in the EP being 45.72 times higher than in the MP. 

The lack of bed number and the absence of justification for not administering the medication also represented the risk factors most present in the EP. Accordingly, adaptations to the electronic system could easily resolve the matter, contributing to the prevention of errors and increasing patient safety. 

The variables, presence of alterations or suspensions were reduced in the EP, configuring another factor that favors safety, since the lower the number of modifications, the lower the risk of errors.

At first, the schedule is set by the nurse and the system maintains it until someone changes it. However, a probability of errors was often verified due to, for example, duplicated therapies with the use of two medications for the same purpose, scheduled for the same time, as well as medication interactions. As the system has the support for the clinical decision resource, there is the possibility of easily eliminating errors of this nature if all the resources are used. However, even with this weakness, the risk of the presence of scheduling with error/erasures in the MP was still 6.05 times higher than in the EP. 

With regard to incomplete scheduling, there was a 3.43 times greater chance of this being present in the MP than in the EP. Therefore, there was a significant increase of these variables in the electronic prescription, demonstrating another of its benefits. Medications administered without checking, also presented in Table 1, is another worrying factor, as this increased from 31.1% in the MP to 79.2% in the EP, constituting an 8.41 times higher risk.

The absence of justification for not carrying out the checks also increased in the EP, which is attributed to the ease of noting the reason for not checking in the MP. Furthermore, the fact can also be associated with the distance of the computers from the bed of the patient, which increases the chance of forgetting to record this information, or with the lack of ability of professionals with the electronic system, so that the check is often performed incorrectly. 

The following constitute factors that did not contribute to the prevention of medication errors: "presence of medication without checking" and "lack of justification for not administering the medication". 

Table 2 shows the information of the medical prescriptions according to the lack of presentation, route, dilution, frequency, presence of prescription with brand name and inappropriate acronyms and abbreviations.

**Table 2:** Distribution of the medical prescriptions according to the lack of presentation, route, dilution, frequency, presence of prescription with brand name and inappropriate acronyms and abbreviations. Brasilia, FD, Brazil, 2013

Essential information on the medical prescription	Manual n^*^ (%)	Electronic n^*^ (%)	P value^†^	OR^‡^ gross (CI^§^ 95%)
Illegible	88(4.3)	-		
Lack				
Form of presentation	213(10.5)	41(2.0)	<0.000	5.68(4.07-7.99)
Route	19 (0.9)	8(0.4)	0.051	2.38(1.04-5.46)
Form of dilution	1442(71.1)	453(22.3)	<0.000	8.56(7.43-9.87)
Frequency	51(2.5)	6(0.3)	<0.000	8.69(3.72-20.30)
Prescribed medications with brand name	2016(99.5)	639(31.5)	<0.000	96.57(53.58-174.0)
Inappropriate acronyms and abbreviations	2022(99.8)	1767(87.2)	<0.000	28.28(11.86-67.42)

* n=2027.

† p-value.

‡ OR Odds Ratio.

§ Confidence Interval

Regarding the risk factors "lack of form of dilution", "lack of route", "lack of frequency" and "lack of presentation", there was a reduction in all. The possibility of data not being present on frequency, form of dilution, presentation of the medication and route of administration in the MP was significantly higher than in EP. Regarding this aspect, the implementation of EP enabled a safer prescription (p<0.000) (Table 2). 

The risk of prescriptions being issued with the brand- was higher name in the MP than in the EP (p<0.000). Inappropriate acronyms and abbreviations were found in 100% of the prescriptions, and in the medical and nursing outcomes. Often, abbreviations or acronyms are used in order to save time, however, this is a risk factor, since they may be erroneously interpreted by health professionals. 

When used, abbreviations should follow standardization to facilitate their understanding. However, at the study site, an absence of standard abbreviations and acronyms was observed. The risk of using abbreviations was higher in the MP than in the EP.

According to Table 2, all the risk factors analyzed were enhanced with the electronic system, especially concerning acronyms and abbreviations.

Table 3, below, provides information about medications found in the medical and nursing outcomes.

**Table 3:** Distribution of information about medications in the medical and nursing outcomes. Brasilia, FD, Brazil, 2013

Information about medication in the outcomes	Manual n^*^ (%)	Electronic ^*^n (%)	P value^†^	OR^‡^ gross (CI^§^ 95%)
Observations in the medical outcomes	326(16.1)	1114(55.0)	<0.000	6.36(5.49-7.37)
No	1701(83.9)	913(45.0)		
Suspension in the medical outcomes	123(6.1)	102(5.0)	0.085	1.09(0.97-1.24)
No	1904(93.9)	1925(95.0)		
Prescription in the medical outcomes	448(22.1)	411(20.3)	0.083	1.05(0.98-1.13)
No	1579(77.9)	1616(79.7)		
Observations in the nursing outcomes	340 (16.8)	533(26.3)	<0.000	1.77(1.51-2.06)
No	1687(83.2)	1494(73.7)		
SOS Medications^||^ in the nursing outcomes	135(6.7)	184(9.1)	0.003	1.39(1.11-1.76)
No	1892(93.3)	1843(90.9)		

* n=2027.

† p value.

‡ OR Odds Ratio.

§ Confidence Interval

||SOS= medications "if necessary" or "in case of necessity".

The registration of complications and/or observations about medications in the medical and nursing outcomes represents another source, in addition to the prescription, to obtain information. This variable can affect patient safety in the medication process.

It appears that after the implementation of electronic medical records at the hospital where this study was conducted, there was an increase of observations relating to medications in the medical and nursing outcomes. Medical records regarding the medications also became more frequent with the electronic medical record. The risk of occurrence of drug information in the medical outcomes in the EP was 6.36 times that that in MP (p<0.000), as well as in the nursing outcome. The registration of SOS medications in the EP was greater than in the MP. It was verified that computerization favors the records of the medical and nursing staff professionals. 

## Discussion

Medication errors can cause major health problems, with relevant economic and social repercussions that, in a certain way, directly affect the lives of patients and the health professionals and institution, as well as prolong the hospitalization period and affect the treatment^1^ ^-^ ^2^ ^,^ ^4^.

The EP is one of the main measures to prevent medication errors^18^. Studies have shown the possibility of a significant reduction of serious errors by implementing this system, with advanced support for the clinical decision. Accordingly, the results of this study converge with those of other investigations with regard to improving patient safety and reducing risk factors for such situations^19^ ^-^ ^21^.

The systems that support clinical decision are more complete and offer suggestions as to the route of administration and any correction in the drug dose and frequency values. In addition, other more complex systems cover checks for allergies, laboratory test results, drug interactions and provide clinical protocols to support the prescriber^21^ ^-^ ^22^. 

These tools can also improve the process for safer medication administration, using technology, such as smart infusion pumps, computers at the bedside and medication administration system with barcodes. 

In particular, computers at the bedside, in association with the computerization of the system, make the registration faster, reducing the time spent completing documentation by 30% and reducing potential faults^22^. The use of bar codes reduces the occurrence of errors by 93% and ensures high-conformity between what is prescribed and what is administered, also allowing the integration of the electronic system in the handling of utensils. Smart infusion pumps, present in 41% of American hospitals, show good results in the reduction of medication errors, by means of audiovisual alerts when presented with incorrect orders, inadequate dose calculations or programming errors^23^.

The results of this study demonstrate that the illegibility risk factor is virtually eliminated with EP, similar to that found in other national and international studies, which showed^24^ 4% of illegible prescriptions^9^ ^,^ ^21^ ^,^ ^25^. It should be noted that prescriptions that are difficult to understand hinder the practice of nurses and technicians and enhance the risk of errors, thus compromising patient safety. In this context, the EP ensures legibility and integrity of prescription fields, reducing transcription errors and facilitating the tracking of the prescriptions. Furthermore, when the system provides support for the clinical decision, it can improve the prescription and give more transparency to the communication process and reliability for the contents. 

The EP also contributed to the presence of essential information for the prescription (route, dilution, frequency), in that the absence of such data can cause problems at the time of preparation, dispensing and administration of the medication for the patient. To provide all the elements of information is, therefore, essential for a safe prescription.

Other studies show incomplete or inaccurate information as one of the main causes of medication errors, as well as the absence of data on the date and route^10^.

To enter the name of the active ingredient is a procedure considered obligatory by law in the public services of Brazil (Law No. 9. 787, of February 10, 1999), determining that, in these spaces, it is obligatory to prescribe the medications by the generic name. This is a procedure for reducing the exchange of names of similar drugs, as the brand names change from one geographic region to another. Traditionally, the use of the brand name was used in the MP, which can lead to errors. Now, with the introduction of the electronic prescription, this possibility has been reduced, since the name of the active ingredient is used^10^ ^,^ ^21^.

A study comparing manual and electronic prescriptions showed that the frequency of errors decreased from 18.2% with the MP to 8.2% with the EP. The greatest reductions were seen in adjusted probabilities of errors regarding illegibility (97%), the use of inappropriate abbreviations (94%) and the lack of information (85%). In this study, a 57% reduction was observed in the adjusted probability of errors that did not cause harm^20^. 

As in other studies, the benefits of the electronic prescription observed in this study are linked to the fact that it is a technology used to facilitate and ensure the safest prescription of the medications^21^. However, if it is not used properly, it can not achieve these goals. Thus, it is understood that the electronic prescription, by itself, does not eliminate the possibility of medication errors. 

Among its disadvantages, it is worth mentioning that this is a complex project that is still expensive, which restricts the number institutions that can adopt it, even in the USA^3^. Some faults observed in the system can configure another disadvantage: Repetition of prescriptions from previous days, without review, and information entered incorrectly^10^ ^).^ These practices can negatively affect the safety of the medication process and therefore require intervention^21^. 

Finally, the risk factor of erasures should be highlighted, which is reduced with the EP, thus providing more security to the medication process. In agreement with other studies^10^ ^,^ ^21^, the lack of registration of the nursing team regarding administration of the medication was another problem identified in this study, which, even with the implementation of EP, has not been solved. Failure to check medications is something usual, however, it is believed that with adjustments to the system its occurrence can be reduced.

Limitations in the present study are related to the use of secondary data and difficulty of access to the patient record manuscripts. The study analyzed the prescriptions generated by the electronic system and did not include the analysis of its structure and functionality or of its acceptance and the interaction of professionals with the system.

## Conclusion

The implementation of a electronic prescription system is associated with a reduction of risk factors for medication errors. Elimination of illegibility is an inherent aspect of the electronic prescription process, which also minimizes the use of inappropriate abbreviations, erasures and lack of information. The improved risk factors (n=9) with the implementation of the EP relate to the scheduling, changes/suspensions, handwriting, erasures, date and time updated, registration number and allergies registration. The factors that were worse (n=4) with the EP were: presence of medications without checking, justifications for not administering, name and CRM of the physician and bed number, although these are considered easy to resolve with system changes. 

Studies of this type contribute to the development of incentive policies for patient safety and investments in this area, as well as preserving the health professionals and protecting the patients. The contributions of the study results are applicable to the context of the institution field of study, especially for the improvement of electronic prescription practices with regard to the reduction of risk factors for medication errors. 
